# Supplementary material for: Scalable and accurate method for neuronal ensemble detection in spiking neural networks
Source: PLoS One. 2021 Jul 30;16(7):e0251647. doi: 10.1371/journal.pone.0251647 (PMC8323916; doi:10.1371/journal.pone.0251647)
Supplement: S1 Appendix — Link to the Codes repository and the details of the retinal experiments with their respective pre-processing. (PDF) [file pone.0251647.s001.pdf]

Supplementary Appendix

Code

The GUI and the codes used to validate the method using synthetic data can be found at `https://github.com/brincolab/NeuralEnsembles`.

Animals and RGC Recordings

The experimental mice were maintained in the animal facility of the Universidad de Valparaíso, at 20–25 °C on a 12-h light-dark cycle, with access to food and water *ad-libitum.* These recordings were performed for other experimental purposes, and for the present work only one recording was used. The corresponding methods of MEA recording have previously been described [1]. In brief, animals were euthanized under deep isofluorane or halothane anesthesia and both eyes were extracted. Then, one of the extracted retinas was diced into quarters while the other was stored in oxygenated (O_2 95 % CO_2 5 %) AMES medium at 32 °C in the dark for further experiments. The same AMES media was used for continuous perfusion during extracellular recordings. For MEA recordings (MEA USB-256, 20kHz sample, Multichannel Systems GmbH, Germany), one piece of retina was mounted onto a dialysis membrane then placed into a ring device mounted in a traveling (up/down) cylinder, which was moved to contact the electrode surface of the MEA recording array. Data were processed off-line using the Spiking-Circus spike sorting algorithm [2] with default parameters.

Visual Stimuli

Visual stimuli were generated by a custom software created with PsychoToolbox (Matlab) on a MiniMac Apple computer and projected onto the retina with an LED projector (PLED-W500, Viewsonic, USA) equipped with an electronic shutter (Vincent Associates, Rochester, USA) and connected to an inverted microscope (Lens 4x, Eclipse TE2000, NIKON, Japan). The image was conformed by 380 x 380 pixels, each covering $5\mu m^2$. To estimate the RGC receptive fields, a checkerboard stimulus (visual white noise) with a block size of $50\mu m$ was presented at a rate of 60 Hz for 20 mins, with each block independently taking 0 or 255 (max value) in the pixel value scale. To classify the RGC, a green ON-OFF light stimulus was presented, where each part lasted three seconds, repeated 21 times. For the classification analysis, the first trial was discarded.

Automated RGC classification

RGCs were automatically classified as ON, OFF, ON-OFF, and Null depending on their preference to the light stimulus, using the statistical approach presented in Ref [3]. We computed the peri-stimulus time histogram (PSTH) for each RGC and compared the maximum activity in the ON and OFF part of the stimulus. Then, we set a threshold based on the average of the PSTH plus 2 *s.d.*, and if only the maximum in the ON (OFF) part was above this threshold, we considered this RGC as ON (OFF); if both maxima were above the threshold, we considered the RGC as ON-OFF. Otherwise, the RGC was classified as Null due to its lack of preference for the stimulus.

RGC receptive fields estimation

The spike-triggered average (STA) of each RGC, defined as the average stimulus preceding a spike, was computed by the reverse correlation method using the checkerboard stimulus (see Methods subsection) aggregating the 18 frames previous to any emitted spike in a matrix as in Ref [4]. This STA matrix was decomposed using SVD, which estimates the temporal and spatial components of the receptive field, where the former represented the average stimulus fluctuation previous to a spike. In contrast, the latter represented the preferred location of the RGC in the stimuli space. Then, an ellipse was fitted to the spatial component to estimate the RGC receptive field.

Referencias

[1] Angelina Palacios-Muñoz, Maria J Escobar, Alex Vielma, Joaquín Araya, Aland Astudillo, Gonzalo Valdivia, Isaac E Garcá, José Hurtado, Oliver Schmachtenberg, Agustín D Martínez, and Adrian G Palacios. Role of connexin channels in the retinal light response of a diurnal rodent. *Frontiers in Cellular Neuroscience*, 8(August):1–13, 2014.

[2] Pierre Yger, Giulia L.B. Spampinato, Elric Esposito, Baptiste Lefebvre, Stéphane Deny, Christophe Gardella, Marcel Stimberg, Florian Jetter, Guenther Zeck, Serge Picaud, Jens Duebel, and Olivier Marre. A spike sorting toolbox for up to thousands of electrodes validated with ground truth recordings in vitro and in vivo. *eLife*, 7:1–23, 2018.

[3] Stephen Carcieri, Adam Jacobs, and Sheila Nirenberg. Classification of retinal ganglion cells: A statistical approach. *Journal of neurophysiology*, 90:1704–13, 10 2003.

[4] E J Chichilnisky. A simple white noise analysis of neuronal light responses. *Network-computation in Neural Systems*, 12:199–213, 2001.
