# Supplementary material for: Scalable and accurate method for neuronal ensemble detection in spiking neural networks
Source: PLoS One. 2021 Jul 30;16(7):e0251647. doi: 10.1371/journal.pone.0251647 (PMC8323916; doi:10.1371/journal.pone.0251647)
Supplement: S1 Table — (PDF) [file pone.0251647.s002.pdf]

Parameters		
Parameter	Description	Default Value
npcs	Number of principal components considered in the clustering.	6
dc	Percentage of total points that are considered as nearest neighbors of centroids in the clustering algorithm [43].	0.02
minspk	Minimum number of spikes in a single population time bin to be considered for calculations.	3
minsize	Minimum number of core-cells to consider an ensemble.	3
cent_thr	Percentage (%) of confidence level used to fit the δ vs. ρ curve in clustering algorithm [43].	99.9
nsur	Number of simulations of artificial data to generate chance level in the task of finding core cells.	100
prct	Percentile (%) to use a threshold of the random distribution in the task of finding core-cells.	99.9
inner_corr	Threshold for average within-cluster correlation in terms of the standard deviation of the population pairwise correlations.	0

Table 1. Method parameters with their description and default values.
