# Supplementary material for: Scalable and accurate method for neuronal ensemble detection in spiking neural networks
Source: PLoS One. 2021 Jul 30;16(7):e0251647. doi: 10.1371/journal.pone.0251647 (PMC8323916; doi:10.1371/journal.pone.0251647)
Supplement: Attachment — Submitted filename: PlosOne_Reply_to_reviewers.pdf [file pone.0251647.s003.pdf]

Reply to Reviewers: PONE-D-20-33389 *Scalable and accurate method for neuronal ensemble detection in spiking neural networks*

Rubén Herzog, Arturo Morales, Soraya Mora, Joaquin Araya,
María-José Escobar, Adrian G. Palacios, Rodrigo Cofré

January 29, 2021

We would like to thank the referees for their careful reading and pertinent concerns, as they have allowed us to improve our manuscript. We have prepared a revised version of our manuscript, which includes all the reviewer's observations. Bold fonts are used for the referee's comments and italics fonts for our reply. The text added or corrected is printed in red in the new version of the manuscript.

<u>**Reviewer 1**</u>

1) In this paper Scalable and accurate method for neuronal ensemble detection in spiking neural networks , the authors are presenting a new method to identify groups of similarly behaving neurons, and the temporal sequences of these activations. Using both real experimental and synthetic data, they show how a method based on dimensionality reduction combined with a robust density based clustering can identify pattern of activity shared by numerous neurons. Overall, I find the article easy to read and to follow. The methodology is clear and the results well explained. However, I have several concerns before I could recommend the publication of the manuscript.

We thank the reviewer for the positive feedback, for finding it easy to read and follow and state that our methodology is clear and the results well explained. We address the reviewer's recommendations next in this document.

Major corrections:

2) My first comment is on the general structure of the manuscript. I think that the experimental validation (with retinal recordings) should come as an illustration of the methods, i.e. should be at the end of the manuscript. The motivation and the scientific questions could remain as they are, but currently, this is a bit odd to start the paper with results performed on real data, before explaining that they are valid because of tests performed on synthetic data. To me, I would switch the two parts, and develop first thoroughly how robust the algorithm is, before applying it to real data

Following the reviewer's suggestion, we have modified the paper's structure, placing our method's validation at the beginning of the results. This modification also allows us to better explain the paper contribution, which relies on the computational method to detect neural ensembles and not characterize them in retinal data. To highlight this contribution, it becomes essential to begin the results showing the method's robustness on synthetic data before validating it on real retinal data.

3) As far as I understood, the methods seeks at finding groups of neurons with similar responses. This is particularly useful in the retina, where we know that the retinal ganglion cells have different functional responses, tiling the visual space. However, I found that this information is not used to assess the quality of the ensemble detection with experimental data. Indeed, if the methods succeed at finding groups of neurons, then when applied to the retina, we should recover different subclasses

of cells (ON/OFF, sustain vs transient, . . .). See for example the classification that has been made in [Baden et al, 2016] with calcium recordings. If this is true, then one could look at the tiling of the receptive fields for the neurons in the distinct ensemble, and assess if they are properly covering the space or not. If yes, then this could be a hint than the algorithmic method is finding an ensemble which is likely to reflect a particular subtypes of cells. If no, then it could also be used as an hint than these ensembles are made of more complicated sub-circuits, mixing different subtypes. This is I guess what has been done by the authors in Fig1 panel B, C, D, but I think this would deserve a dedicated figure, as the current take home message is not clear. I would like to see the Receptive Fields, not colored as function of ON/OFF/ON-OFF/Null, but rather as function of their ensemble (as this is currently done for the PSTHs). By doing so, one could appreciate if the ensembles are tiling the space, i.e. if the method is able to distinguish putative cell subtypes.

To identify and characterize groups of neurons in the retina with similar responses is an exciting application of the method described here. However, as we previously mentioned, this paper's contribution is focused on the methodology to detect neural ensembles and not to understand retinal physiological or functional properties. Nevertheless, we did separate the data showing both the spiking response of the best core-cell of each ensemble (the most correlated cell), and the tiling of that group's receptive fields. This information can now be observed in Fig. 5. The new Fig. 5 only shows the subsequent results, but we did not discuss the findings' implication nor calculate any measure of receptive field tiling. A complete analysis of different neural ensembles encountered in the rodent's retina -and the validation of cell type mosaics- should be addressed in an independent article.

4) Regarding the experimental procedures. How many animals were used to collect the data? When the authors are talking about 319 RGCs during 120s of MEA recordings, is this on a single recording? This comment makes me realize that the tiling mentioned before might not be so obvious, if data are collected across several animals. But if they are, what do the plots in Fig1B corresponds to ? Can the authors explain what these receptive fields maps are, and if data are from a single retina? If, on the other hand, data are from a single retina, why did the authors not reproduce the analysis on various recordings? This would consolidate the (functional) claims on the ensembles, if the number found could be reliable across animals.

We used a single animal and a single retina to conduct our example on retinal ensembles. Fig1 (now Fig 5B) shows the receptive fields on the single retinal patch studied in this article. The explanation of what are the receptive field maps can be found in the methods section. Our aim with the retina example is to show and application of the method, rather than a specific research in the retina. In fact, with this example, we show how to interpret the results generated by our method in a specific physiological context.

5) From an experimental point of view, and still in line with was has been done in [Baden et al, 2016], why the authors restricted themselves to very low dimensional stimuli (ON/OFF light stimulus)? If the goal is to detect ensemble, i.e. cells sharing similar responses, then it might be more interesting to display high-dimensional stimuli, to trigger more variability in the responses, and thus get a closer look on these putative ensembles. The more diverse the responses will be, the more the ensemble might appear (assuming there is approximately 30 subtypes of retinal ganglion cells in the mammalian retina, I would naively assume that there should be much more ensembles popping up).

Our contribution's scope is to present a general scalable and accurate method for neuronal ensemble detection applicable not only to the retina. We used synthetic data to validate our method, and we then present an application example on retinal data. Nevertheless, we did not analyze any particular feature of retinal ensembles, only showing the results obtained for a low-dimensional stimulus (light-flashes). We agree with the reviewer that stimulus diversity increases the number of activated neural ensembles, being an excellent opportunity to study these ensembles' properties. However, it is out of this paper's scope, where we focused only on the methodology to detect those neural ensembles.

6) What is the rationale for discarding responses with low firing rates?

In our experience analyzing retinal data, cells with low firing rates mainly obey the spontaneous activity instead of light-evoked response. With this in mind, we discard neurons with low firing rates to focus on the evoked activity, which better represents the cell's dynamics. Right after the spike sorting, the preprocessing stage includes removing cells with low activity (firing rate $< 0.01Hz$). This stage is not part of the algorithm described in this article.

7) There is something I do not get with the methods: in the discussion, the authors are talking about

the fact that they used "the standard bin size for mammalian RGCs". I guess this bin size is used to bin the spike trains before PCA, but this is not explicitly said in the methods, is it? Or I may have missed it... Anyway, what is the value used in the paper?

The standard bin size for mammalian RGCs is of 20ms. This choice is based in several previous studies that have made this choice based on properties of single and collective spiking neurons. The reviewer is right that the bin size is used to produce the spike trains before PCA, and that need to be mentioned. In the new version of the manuscript this value is indicated in the result section dedicated to retinal analysis.

8) What is the rationale for allowing only one active ensemble per? I know that otherwise, non-linearity are likely to perturb the methods, but this is a very strong assumption that need to be discussed. While it might be true for retinal activation, assuming different stimuli are triggering different subtypes/responses, one at a time, this assumption does not hold in the global context pictured by the authors in the Introduction. Cortical ensembles in vivo might overlap both in time and space. Please comment on that.

The method proposed in this article detects neural ensembles, defined as population vectors containing neurons firing together in a specific time window i.e. a bin. These population vectors have the same dimension as the total number of neurons. The method only detects groups of neurons spatially correlated (i.e. they fire in the same bin) and does not discriminate if the ensemble's core-cells respond differently to another type of stimulus. An in-depth study in the retina response, using stimuli with different statistical structures, could separate between sub-types or between ensembles overlapping in time and space. Nevertheless, as we previously mentioned, this article proposes a fast and scalable method to detect neural ensembles and not to characterize neural ensembles encountered in the retina.

9) As the authors acknowledges themselves in the Discussion, different time bins might affect the results. It would be good to provide an analysis on the robustness of the methods w.r.t. the time bin. One could not expect the results to be the same, but a quantification of the variability is important. Since there is no guarantee that the chosen time bin is optimal experimentally, at last it could give some confidence in the results. This can be explored with the synthetic data generated by the authors.

The method implemented to generate synthetic data fill with 1 or 0 the time bins, without reference to any time width. For real data, the method binarizes the presence (1) or absence (0) of cell activity inside the time bin, encountering different ensembles for different time bins. As the bin size is transparent for synthetic data, there is no manner to provide the analysis proposed by the reviewer. Nevertheless, we could implement this analysis for real data to detect, for instance, if the detected ensembles are preserved at different time scales. However, this analysis relies on understanding retinal physiology being out of the scope of this methodological paper

10) What about time-warping? I know this might be out of the scope of the manuscript, but maybe it would be worth comparing a bit the method proposed here with the ones seeking at the detection of assemblies taking into account the fact that sequences can be time-warped [Williams et al, 2020, Mackevicius et al, 2019]. In the current paper, the authors are seeking at a fixed sequence of activation between ensembles, but this assumption of a fixed bin size should be discussed.

We thank the reviewer for this interesting recommendation. In synthetic data, neural activity is represented in a time-free format where time-wrapping analysis is not feasible. However, time-wrapping could be an excellent tool to evaluate if the neural ensembles detected for real data are comparable at different bin sizes. We expect to have different ensembles for different time bins. In the example shown with retinal data, the choice of the bin size is motivated by previous studies that consider 20ms (10.1113/jphysiol.2010.193888 or 10.1038/nature04701). Nevertheless, time-warping is out of the scope of this article, whose primary motivation is to present a scalable and accurate method to detect neuronal ensembles. The reviewer raises an interesting question for future research. We provide all the codes and a GUI to facilitate our method's use by the scientific community.

11) What are the equivalent of low/medium/high density cases for real data? More precisely, where are the retina data, on this scale? This should be said to enforce confidence in the results, since the low density case seems to be more problematic.

We observed that the retina activity level depends on the stimuli, with different spike densities for spontaneous, artificial, and natural stimuli. In this article, the simulations made with synthetic data used levels accordingly with the retina response for these three types of stimuli. The analysis with real data for the light-flashes stimulus corresponds

to a medium activity level. Following the reviewer's suggestion, this is now indicated in the article.

References

- Williams et al, 2020, Point process models for sequence detection in high-dimensional neural spike trains, https://arxiv.org/abs/2010.04875

- Williams et al, 2020, Discovering Precise Temporal Patterns in LargeScale Neural Recordings through Robust and Interpretable Time Warping,
https://doi.org/10.1016/j.neuron.2019.10.020

- Baden et al, 2016, The functional diversity of retinal ganglion cells in the mouse, Nature,
https://doi.org/10.1038/nature16468

- Mackevicius et al 2019, Unsupervised discovery of temporal sequences in high-dimensional datasets, with applications to neuroscience
https://elifesciences.org/articles/38471

Reviewer 2

In this manuscript the authors introduce a new pipeline for the detection of synchronous cell assemblies in parallel spike trains. The proposed algorithm relies on density-based clustering of population activity patterns. The algorithm performance is assessed on simulated ground truth and then tested on spike trains from in vitro retinal ganglion cells during an ON-OFF light stimulation. I found the simplicity and the scalability (huge problem for the community) of the algorithm the strength points of the proposed manuscript, which I found useful for the scientific community. However, there are a number of major points to address before publication.

We thank the reviewer for your careful review, insightful suggestions and the positive comments about our manuscript. We are grateful for your words about the strengths of our algorithm.

2) Major remarks–1. Assessment of core-cells: Once established the assembly core patterns (centroids) the authors define the core-cells of each assembly by testing the correlation between single-unit activity and assembly signals. This is done by shuffling the temporal sequence of assembly activations. While I found appropriate using bootstrap techniques to assess the correlation significance, in case of time series it is important to preserve the internal autocorrelation of the shuffled signal to avoid false discoveries. The most common, but not exclusive, way of doing so is by first dividing the time series in windows of size comparable to the signal autocorrelation length, and then by permuting the order of such windows [Efron Tibshirani, Introduction to the bootstrap, 1993, Springer]. I, therefore, suggest the authors to address this point and modify the algorithm accordingly. Also, the authors leave the significance parameter of this test as free parameter. This should not be the case, as significance it is not arbitrary, and this parameter should be limited to < 0.05.

The reviewer raises a fair concern. Spike trains auto-correlations in retinal ganglion cells decay very fast to zero, it is around 20ms , which is the time bin we use in the example of the retina (see for example 10.1113/jphysiol.2010.193888 or 10.1038/nature04701.) The synthetic data was generated by a memory-less stochastic process, thus no lag auto-correlations is expected. Throughout the paper, the significance is set by its default value < 0.05. *However, as we believe that depending on the amount of data available, some authors may prefer to set this threshold by themselves, we leave open the possibility that this parameter may be modifiable.*

2. The simulated time series used to test the validity of the method are quite unrepresentative of real biological data: a) they are stationary; b) the ratio between assembly activations and background spikes is extremely high, to the point that (in the low and medium density case) almost all spikes fired by a unit take part to one or the other assembly; c) as mentioned in page 11 lines 295-296, non-assemblies spike patterns are filled with only silent spikes. In these extreme conditions is a bit difficult to assess the algorithm qualities. I would therefore suggest testing the false discovery rate of the method by using a non-stationary background (non-assembly) activity for all units, and a way smaller ratio between assembly and non-assembly activity. In particular, I suggest formally evaluating the false discovery rate (in terms of detected core-units and assemblies) for a non-stationary background activity and: 1) a range of values of total assembly activations; 2) a range of values of percentages of assembly spikes missing (similar in spirit to what done for Fig 3B).

We partially agree with the reviewer. The synthetic data is generated to test out method not necessarily to accurately represent biological data. Indeed, the synthetic data is stationary but this is not a limitation for our method as shown on the retina example that exhibit good performance under a time dependent (periodic) external stimulus scenario. The second limitation mentioned by the reviewer can be adjusted easily in the synthetic data we generated and do not represent a limitation of the method as shown by the example on the retinal ganglion cells where some RGC do not belong to any ensembles. The third limitation mentioned by the reviewer is not an arbitrary assumption. The most frequent spiking pattern in the retina responding to light stimuli is the pattern of only zeros (see figure 5 in https://journals.plos.org/ploscompbiol/article?id=10.1371/journal.pcbi.1003408). Besides, after generating the ensemble activity, the spike train of each neuron is corrupted by adding/removing spikes in order to satisfy a given firing rate. This converts several non-spiking population vectors into vectors with random spikes. Finally, the ratio of ensemble/non-ensemble activity was chosen to approximate the ratio observed in the retina experiment (70%). We consider that our results accompanied by the Matlab codes provide enough evidence and tools for the application of the method in experimental and synthetic data.

3. I imagine that detecting assemblies composed of very few core-units might be challenging for this kind of approach. I would therefore suggest discussing this limitation (if present).

Given that several methods exist today to detect significant pairwise interactions, we chose to focus in high-order population vectors (3 or more spiking neurons in a vector). Our approach is capable to detect ensembles of few core-units as illustrated in the examples performed in the synthetic data.

4. As last step of the algorithm "The inner-cluster mean correlation is compared against a threshold of non-core cell correlation, discarding any cluster below this threshold". This final step is not clearly explained and would benefit of some more detail. How is this threshold chosen?

We thank the reviewer for noticing this issue. In the first section of our revised manuscript, we have added a short subsection explaining in more detail the procedure we use to evaluate the within-cluster average correlation and explain how we compare this correlations with other correlations obtained by chance.

5. The sentence "Among them, only the one based on the correlation between population patterns, and the one based on non-negative matrix factorization, fit with a definition of ensembles." is either false or confusing. I would suggest rephrasing or removing.

We agree with the reviewer. We have removed this confusing phrase.

6. In the discussion, the authors compare their method with other state of the art techniques (Page 13, lines 372-382). I found this paragraph a bit surprising, as a large variety (I would say the majority) of assembly detection techniques can detect synchronous assembly patterns and with no-event related information. I would therefore suggest to rephrase and tone down this paragraph and, maybe, focus more on the method scalability, real point of strength.

Thanks for your comment. Our method and our comparison approach does not rely on any event related information. Our method as several other ensemble detection methods only rely on the spike trains. What we did in the example of the retina is to detect the ensembles only from the spike trains. Afterwards, we analyse their temporal sequence of activation in comparison to the time in which the stimulus was presented to the retina. We realized that for this very simple ON-OFF stimulus the ensembles activation times are highly related to the stimulus, but this is an a-posteriori analysis that is not part of out method of ensemble detection.

Minor remarks

Minor points: 7. Please, define mathematically the quantity "relative error".

We used the formula $(V_O - V_T)/V_T$ *where* V_O *stands for observed value and* V_T *for theoretical value. We include this formula in the main text.*

8. Please, indicate the value of all arbitrary parameters used for each analysis (also for the SDV-based method).

A table with the parameters of our method is now included in the Supplementary Material. The parameters used in the SVD-method are several and can be found in the original scripts made available by the authors in GitHub.

9. There are relatively many parameters that the authors leave free for the user to decide. While user necessities are multiple and have to adapt to diverse datasets, a total arbitrariness of these choices might lead to extreme settings and unreasonable assembly detections. I think it would beneficial for future users to discuss the implications behind each parameter choice (e.g. by showing the consequences of choosing a different number of PCA principal components when many assemblies are present) and to suggest a parameters set "safe" for the users to use.

We thank the reviewer for this observation. We provide a table with the parameters used in out method and its default values.

Reviewer 3

This manuscript proposes an analytical method to detect neuronal ensembles from MEA electrical recordings. The work demonstrates that the method could be used in retinal recordings exposed to ON-OFF light stimuli. The authors validated the method using synthetic data to identify different neuronal ensembles. The standardization of methodologies to identify groups of neurons with coordinated activity that are related to specific experimental conditions is a relevant topic in neuroscience since the simultaneous recordings of neuronal populations has become worldwide available and is growing at a steady pace. The manuscript describes a potential method to identify neuronal ensembles, but several concepts and implementations should be clarified in order to make the method useful for diverse brain structures. Otherwise, the tittle should be changed to highlight that the method is useful mainly for in vitro retinal recordings and specific experimental conditions. Finally, the analysis for the sequential organization of the ensembles needs to demonstrate that sequential patterns of activity could not be identified in random population activity.

We thank the reviewer for his insightful review and for the suggestions on how to improve our manuscript.

1) The definition of what is the meaning of a neuronal ensemble is not clear from the abstract nor the introduction. This is a relevant issue since the goal of the manuscript is to detect neuronal ensembles. It is mentioned that a neuronal ensemble is a group of neurons that fire together (page 1, line 9) but the authors indistinctively call a neuronal ensemble a group of neurons firing together or different groups of neurons firing in sequences. The methodological demonstration of groups of neurons firing together or groups of neurons firing in sequences are two completely different approaches.

We thank the reviewer for raising this critical issue regarding our working definition of neural ensemble. In our work, similar to the quantitative definition proposed by Reviewer 3 in Carrillo and Yuste 2020, we consider a neural ensemble as a set of multi-dimensional population vectors that cluster together in a suitable low-dimensional space. In addition, we specify that an ensemble should have at least one core-cell. Otherwise we consider that cluster as non-ensemble population vectors given its lack of significant correlation in time with any neuron of the recorded population (further details on core-cells on reply to point 2 of Reviewer 3). To clarify a possible confusion regarding ensembles conceived as synchronous firing vs firing sequences, we note that our method clusters population vectors, so the detection of an ensemble imply the detection of the activation times of that ensemble. Then, if two or more ensembles are detected, there will be a sequence of ensembles in time, building up the global ensemble sequence. However, our method does not offer any analytical tool to evaluate the significance of the transitions and robustness of the detected global ensemble sequence. We included a more explicit definition of neural ensemble in the Introduction and change the term 'population pattern' to 'population vector.'

2) Page 2, line 42. What is the meaning of core cells? Does core cells represent the same concept of a neuronal ensemble? In such case the use of core is unnecessary and confusing.

We define a core-cell as a neuron whose spike train is significantly correlated with the activation times of a given cluster of population vectors. See Methods, Core-Cell detection section for the operational and statistical definition. The difference between core-cells and ensemble is that the neural ensemble can recruit cells whose activity is not significantly correlated with the temporal activity of the cluster (non-core), while core-cells are significantly correlated with the ensemble. We recall that the population vectors building up a cluster are similar between them, not equal, so even when the activation times of a cluster are known, there is no a priori knowledge of the presence of core-cells. As an illustration of this difference, let us consider a human assembly, where the leaders/organizers of this assembly would be the core-cells ans of the assembly (each time the assembly gathers, the core-cells are probably present), while the non-core cells randomly attend to this assembly and make part of it, but they are not the core of the assembly. This notion of core-cells emphasizes the stochastic nature of neurons that can randomly participate in diverse neural ensembles without the need to be part of the core-group, making the ensemble greater (in terms of the number of cells), and more robust than the sole activity of the core-group. The importance of the core-cells, as the importance of the leaders/organizers of a human assembly, is that they best represent the activity in time of the ensemble, but they are not the ensemble as a functional whole. To clarify this issue, we have included an explicit definition of core-cells in the introduction, stressing the difference between ensembles and core-cells.

3) Page 3, lines 72-79. The use of the concept of core-cells is not clear. Why "the ensembles as a whole are not a simple inheritance from their corresponding core-cells" if the authors define a neuronal

ensemble as the core-cells in the previous page.

Considering the aforementioned clarification we stress the fact that the ensemble activation times can be different from the activation times of their corresponding core and non-core cells. Core-cells are significantly correlated with their ensemble' activation times, but not perfectly correlated, so the activation of a given ensemble cannot be entirely determined by the activity of its core-cells. We mention in this issue on the corresponding section.

4) Page 3, line 83. It is not clear what is the meaning of scalable. Does it mean different brain areas or different orders of magnitude in the number of recorded neurons? Or different lengths and sizes of the data?

We thank the reviewer for pointing pout this issue. We refer to different lengths and sizes of the data. Our method is mainly for spiking neuronal network analysis, but it can be applied to any multi-variate binary dataset.

5) Page 3, lines 95-96. In the example shown in figure 1A, it is clear that ON and OFF early responses have different properties as length and amplitude. In particular, the approach used in the manuscript (PCA) will enhance such differences in the low dimensional projection of the data. However, in many other conditions and brain structures the activity of different ensembles could have the same properties limiting the use of PCA to separate such subtle changes. The authors should show that ensembles with the same overall characteristics of population activity could be detected with PCA. Otherwise, the manuscript should clarify that this method is useful for retinal recordings.

The reviewers raise a fair concern, however, the late ON and OFF response are similar in length and magnitude and our method still detect them as different neural ensembles. Indeed, in the example in the new Figure 2 the ground truth ensembles differ in magnitude, while the rest of the analysis on synthetic data was performed with ensembles with the same magnitude. Based on the aforementioned facts, we consider that our method is general enough to be applied to other neural tissues where population vectors can have different and/or similar characteristics.

6) Figure 1A. From the figure Bottom it is hard to see if many ensembles could be active at the same time? If this is the case, then the definition of a neuronal ensemble becomes critical for the claims of the manuscript. Because, in that case several combinations of different groups could potentially represent different neuronal ensembles.

Following the aforementioned definition of ensembles as multidimensional population vectors, our method, at its current state, is unable to distinguish if an ensemble is composed by sub-ensembles, or to reverse engineering the ensembles to find their underlying generative process. The decomposition of the detected ensembles into sub-ensembles or the mechanism, giving rise to the detected ensembles are out of the scope of our method, which is designed to detect those population vectors that can be clustered together, and whose activation times are correlated with at least one neuron of the recorded population.

6) Figure 1B. The labeling is confusing because it is mentioned before that four different ensembles are expected. What is the meaning of ON-OFF?

We agree with the reviewer that the use of ON-OFF for stimuli and cell types can be confusing, but is the traditional way in which neurons in the retina have been classified according to their preferences to a light stimulus. On the one hand there is the ON-OFF stimulus, which is a periodic light-dark cycle. On the other hand, there is the ON-OFF cell type, which corresponds to a cell which preferentially fires under light and dark stimuli. That is why the legend in Figure 1B specifies 'ON-OFF RGC' to distinguish from ON-OFF stimulus.

7) Page 5, lines 148-150. It is not clear what measurement was used to reach the conclusion that the ensemble tuning preference cannot be completely derived from their core-cells.

The reviewer is right, we did not make quantitative measurements that allow us to conclude what was claimed in the previous version of the manuscript. Indeed, we did a qualitative comparison between RGC PSTH and ensemble PSTH because our scope is not to deepen into retinal ensembles, rather, we wanted to test the method on an experimental set-up where simple hypothesis can be tested. We changed the last paragraph of that section, and clarify that our results suggest (not conclude) the presence of ensembles in the early visual system, and that their activity as a whole functional unit is different from the function of their corresponding core-cells.

8) Even though the method is intended for a broad neuroscience community. It is important to mention in the results how the spikes were extracted and how the ON and OFF cells could be identified.

We thank the reviewer for the suggestion. We mention that in the supplementary methods section, but now we included a small mention in the results as well. **9) Page 5, line 156. It should be indicated in the results what is the meaning of a fixed number of spikes.**

We thank the reviewer. We make it clearer in the new version.

10) Page 5, line 159. It is mentioned that only 3 to 6 principal components are used. The authors should indicate in the results section what is the criteria to select 3, 4, 5 or 6 principal components.

We actually used 6 PC, so now it is clearly stated in the article. In addition, we add a small mention on how to choose the number of PCs based on the cumulative explained variance and the cut-off the of the eigenvalue spectrum of the PC decomposition.

11) Page 8. Section Detection of ensembles on synthetic data. To test the method the authors generated synthetic data that resembles the data showed previously from retina. As I mentioned before the manuscript shows that the method is ad hoc for retina recordings but not other brain areas.

We thank the reviewer for this fair concern, however it is not clear to us what is the question/suggestion/criticism here. We generated data with 3 different densities, which resembles different levels of spiking activities. In general, neurons with a high firing rate reach $\sim$ 300 Hz, which corresponds to a firing probability of 0.3 using a bin size of 1ms. The densities that were chosen range from very low firing rates to firing rates compared to the above mentioned one. See the distributions in Fig. 2 panels A-D where the firing rate of each neuron is shown. We consider that our method is not ad-hoc for retinal recordings, instead, as stated in the title, is designed for spiking neural networks, i.e. simultaneous parallel multi-unit action potential recordings.

12) Page 8, lines 195-206. The manuscript introduced here the detection of sequential activity patters. But the criteria to define sequential patterns of activity and their statistical significance were not mentioned anywhere. Such criteria should be included otherwise should be removed from the paper.

This is a fair concern. As stated on the reply to the first point of Reviewer 3, our method does not offer any analytical tool to evaluate the statistical significance of the detected sequences. We did not detected sequential activity patterns, what we detected is the activation times of each ensemble which, together, yields the global activation sequence. This global activation sequence can be pictured as a string, where the left-most symbol is the first time point and the right-most symbol the last time point; time evolves from left to right, and the symbol on each time point corresponds to one of the detected ensembles or none of them. We now clarify this in the new vesion of the manuscript.

13) Pages 8-9. To evaluate and compare the performance of the method the manuscript compared the results obtained with synthetic data using the current method and a previously published method (SVD-based). As I mentioned before the authors showed that their method is useful for data with specific characteristics so it is expected that the performance will excel other methods. On the other hand, the method compared (SVD-based) was used for optical recordings whereas the current method analyzed electrical recordings. The difference in the temporal resolution of the data could also bias the results. The authors should compare their method against other method applied to electrical recordings or discuss the limitations to apply it to data obtained with lower temporal resolution.

This is related to point ***11***. *Our method works on binned data, so even if optical recordings have a different temporal resolution than spiking data, in the end, the data to be analyzed both in our method and in the SVD-method is a binary matrix. If activity of neurons recorded using optical methods (once properly binned) yields activation probabilities similar to those observed in spiking data ($\sim [0.05, 0.4]$), the synthetic datasets used here should resemble both optical and electrical recordings and, thus, both the SVD-based and our method should be able to detect the ground-truth. Regarding comparing to other methods, we researched the available implementation on the web, and we did not find any implementation (besides SVD-based) that clusters population vectors into ensembles and find their core-cells. Given that our scope was not to implement other methodologies, we reduced our analysis to the already implemented methods currently available.*

14) Page 10. Synthetic Spike Trains section. It is not clear why the authors used a Bernoulli process to

define the activation sequence of each ensemble. A Bernoulli process define a series of random events that are independent whereas the sequential activity of neuronal ensembles has been shown to be time dependent.

Our method does not take into account the temporal correlation between ensembles for the clustering step, so there is no need to include a time-dependent process in the generation of the activation sequences.

15) Page 11. Feature extraction using PCA section. The authors should be consistent in the terminology they used along the manuscript. This section mentions that spike patterns with less than 3 spikes were discarded. What is a spike pattern? Is this a population pattern or a single cell pattern? Why 3 spikes?

We corrected the terminology, and now we use population vectors to refer to the synchronous activation of neurons. We used a minimum of 3 spikes in order to focus in high-order interaction rather than pairwise.

16) Page 11. Previously the manuscript mentioned that 3 to 6 principal components (page 5, line 159) were used but now they mention that 4 to 7 principal components were used (page 11, line 309).

Corrected. A small mention of how to inform the choice of the number of PCs is now included.

17) Page 11, line 313. What is close pattern' clustering?

Corrected. We meant clustering population vectors based in their distance in the Distance matrix.

18) Page 12, Centroid detection section. It is not clear from the text how the clustering is performed. Is it a hard or a soft clustering?

It is a hard clustering and now we make the clustering section clearer. The implementation details can be checked in the GitHub and in the original publications cited in the corresponding section in the article.

19) Did the authors remove the points that couldn't be clustered in the low dimensional space and then perform the clustering again? The main limitation of clustering high dimensional data is that all the points will appear in the low dimensional representation even though they represent unique events.

Once projected in the PC space, all the population vectors are assigned to a cluster. The vectors that belong to clusters without core-cells are considered as non-ensemble vectors.

20) What is a closest centroid? The manuscript didn't mention any measurement for that.

The distance matrix between vectors allows the computation of the centroids, which in turn yields the distance to the closest centroid in the PC space for each vector.

21) Page 12. Core-cell detection section. This section described the procedure to detect core-cells. However, from the text it is not clear why these core-cells are important or what is the difference between an ensemble and the core-cells of an ensemble. Is it the same?

This related to the point 3, where we gave the definition of core-cells (now in the new version of the manuscript). Their importance is that they best represent the ensemble temporal activity, their presence suggest functional coupling between ensembles and single neurons (i.e. multi-scale coupling), as exemplified with the case of the retina.

22) Page 12. Ensemble selection criteria. The authors mention two criteria for ensemble selection. But from the text it is not clear how to choose such parameters. This is important for anyone interested in applying the method to their own data.

Those criteria now are made clearer in the new version of the manuscript. These are free parameters of the GUI.

23) Page 13, line 386. The SVD-based method from the reference cited (16) didn't extract a temporal sequence of ensembles as mentioned in the text.

We agree with the reviewer. We meant temporal activation of ensemble rather than temporal sequence. The temporal activation of ensemble can be obtained with the SVD-method.

24) The authors should compare different bin sizes as they mentioned in the discussion. This is very important for the broad use of the method.

The method uses as input a binary matrix, so the binning is not part of the method. For the retina case, we use the standard bin size for retinal recordings. For the synthetic data no bin size was used because we generated the binary matrix directly.

Minor remarks

1) Page 2, line 39. It is not clear from the text what is the meaning of "one-time bin of the spike train".

Thanks for pointing out this potentially confusing phrasing. What we mean by "one-time bin of the spike train" is a spike pattern with no memory, i.e a column in the spike train matrix build as usual Neurons $\times$ Time-bins. In order to avoid confusion we rephrase as "the synchronous binary population vectors" and erase "one-time bin of the spike train".

2) Page 3, lines 66-67. Revise "...when confronted to changes light intensity"

Thanks for this remark. We change the phrase to "...responding to changes light intensity"

3) Page 3, line 88. What is parallel recording? Does it mean simultaneous?

Yes, we mean simultaneous. While the words "parallel recordings" has been used before in this context simultaneous recording is more clear. Following the reviewer suggestion, we changed from parallel recording to simultaneous recording.

4) Page 4, lines 114-115. The conclusion of this sentence is not clear since the data only shows ON and OFF responses. What does it mean without any stimulus-related information?

We mean that our method only analyze binary spike train responses and does not rely on any stimulus-related information. Once the ensembles are identified we assigned to each spike pattern in the spike train their corresponding neuronal ensemble. When this is done we observed that the method clearly differentiate ensembles associated to the ON activity and OFF activity.
