# Supplementary material for: Scalable and accurate method for neuronal ensemble detection in spiking neural networks
Source: PLoS One. 2021 Jul 30;16(7):e0251647. doi: 10.1371/journal.pone.0251647 (PMC8323916; doi:10.1371/journal.pone.0251647)
Supplement: Attachment — Submitted filename: PlosOne_Ensembles_reviewer_response_R2.pdf [file pone.0251647.s004.pdf]

Reply to Reviewers:
PONE-D-20-33389
Scalable and accurate method for neuronal ensemble detection in spiking neural networks

Rubén Herzog, Arturo Morales, Soraya Mora, Joaquin Araya,
María-José Escobar, Adrian G. Palacios, Rodrigo Cofré

April 16, 2021

Bold fonts are used for the referee's comments and italics fonts for our reply. The text added or corrected is printed in red in the new version of the manuscript.

1) First, as visible from Fig.5A retinal ganglion cells have a clear autocorrelation. Secondly, and most importantly, the goal of the paper is to provide the scientific community with a tool to detect cell assemblies in experimental data. It is well established that neuronal time series are non-stationary and autocorrelated, ignoring this fact leads to a wrong significance assessment and false discovery rates. The fact that the synthetic data used in the paper are stationary just means that they were not suitable to test the algorithm (and this is why in point 2 of my original review I asked the Authors to test the algorithm on more realistic time series). I am very surprised that the Authors decided to neglect this fundamental request, both because of its major importance and because it can be easily fixed (as explained in point 1 of my original review). As side note, a significance value above 0.05 means that the result is non-significant. Increasing the alpha value (that is the threshold for significance) can not solve problems of low statistical power.

First, as we stated in response to the first Major Remark raised by the reviewer in the first round, the selected bin size (20ms) produces autocorrelation functions decaying fast. In just one time step, the autocorrelation functions decay to half (or less) of its maximal value. In fact, on average, after just one time bin, the autocorrelation function decays more than 80%. (see Figure 1 below). That is mainly why several researchers consider that successive spike patterns are time-independent (see Schneidman, E., Berry, M., Segev, R. et al. Weak pairwise correlations imply strongly correlated network states in a neural population. Nature 440, 1007–1012 (2006). https://doi.org/10.1038/nature04701). We added a comment on the Discussion section regarding existing methods to test the correlation between spike trains preserving some temporal features as inter-spike intervals or autocorrelation. We acknowledge that this can be easily implemented in our code, but we chose the most general case, where no temporal structure of the spike train is considered.

Second, undoubtedly the assumption of stationarity is a limitation of our approach, as it is for many other works in the field of spike train analysis. In fact, the responses to ON-OFF stimulus-induced non-stationary activity both in short and long timescales: In short time scale, the change in the light intensity induced a dramatic change in the firing rates; and, on the other hand, in the long time scale, the average population activity decays around 15% from the beginning to the end of the recording. Despite this limitation, the method can detect -with no prior information about the stimulus presentation time- retinal ensembles that are tightly tuned to specific features of the stimulus (ON light or OFF light), as expected for neural population activity in the visual system. In addition, the remarkable performance with synthetic data suggests that the closest to stationarity the data is, the more reliable our method performs, as for most of the statistical methods that assume data stationarity.

Finally, as we explain in response to the reviewer's first Major Remark, the significance threshold for core-cell detection is set to 0.05. Consequently, any correlation value whose probability of being observed is less than 0.05, given the null

hypothesis, is considered significant. This threshold value is a free parameter in the GUI, so the user can freely change it according to his/her criteria. Using a threshold above 0.05 is a user's decision (which could be eventually justified) and not a limitation of our method.

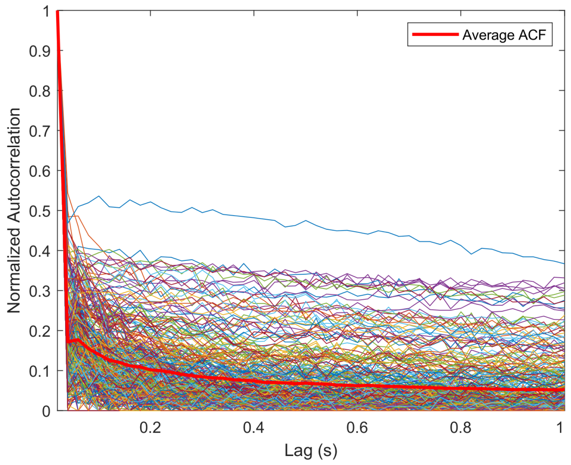

Figure 1: Autocorrelation function (ACF) of recorded RGCs (thin lines), and average of the ACF (red thick line)

2) The purpose of the synthetic data is exactly to reproduce all characteristics of the biological data which might affect the reliability of the algorithm. The algorithm is presented as a general methodology to test cell assemblies and not to test exclusively retinal ganglion cells (if it was explicitly aimed to test retinal ganglion cells, this should be clearly stated and the Authors would still have to change their synthetic data, since the data in Fig. 2 don't have the same characteristic of autocorrelation of those shown in Fig. 5A). Of course I understand that all algorithms have some limitations, but such limitations have to be tested and addressed in the manuscript to inform future users. In this manuscript this assessment is very poor and misleading.

As stated previously, at the chosen bin size, RGC's show a fast decay on their autocorrelation function. This bin size is typically selected in the range of 10-30ms with the purpose of yield fast-decaying autocorrelation functions so temporal dependencies can be neglected. We consider that it is not possible to reproduce all the characteristics of biological data, so we chose to generate the simpler and more general feature of spiking populations: the distribution of firing probability. More sophisticated extensions of our method will be eventually required for particular scenarios, so we encourage anyone interested in context-specific improvements to collaborate with us or modify the already available codes.

3) Again, the limitations of the method have to be tested and described. I might have missed something but to my understanding it has not been studied how the assembly discovery rate varies as function of the assembly core size. Clarifying this point will be of great relevance for future users who might interpret why they can not find assemblies if the number of core cells is much smaller of the whole population number (if this is true, it should be indeed be tested).

Please note that in the revised version of the manuscript (Fig. 4, Detection performance over a wide range of spike-train parameters), we tested the method's performance with synthetic data where both the number of ensembles and core-cells changes. We decided to use the correlation as a measure of model performance: in the case of ensemble activation sequence, we computed the correlated between the ground truth sequence and the detected one, and in the case of core cells, we computed the correlation between the binary N-dimensional vector of ground-truth core-cells (n=1 if n is core, 0 otherwise) and the detected one. Despite the existence of several ways to measure the detection performance, we consider that correlation is enough to illustrate the flexibility of the method. In our case, the correlation values were highly correlated with the true positive rate ones, so we decide to summarize the results using the correlation measure.

4) Thanks for the clarification. Since the threshold for ensemble selection comes from the average pairwise correlation of the whole population, would this create a problem if e.g. all units of the tested sample take part in one global-assembly? Would this whole population-assembly be detected?

We thank the reviewer for the comment. As shown in Figure 4 in the current version of the manuscript, our method's performance is highly impaired when the number of ensembles or the number of core cells is small. So, the case described by the reviewer is explicitly shown as a method's limitation in the main results.

6) I had understood that the presented method does not rely on any event related information. Point 6 of my old review referred to the Author's sentence "Despite the usefulness of these methods in their context, our analysis is more general. It relies on grouping the population spiking patterns with no event-related information . . . " This sentence hint at the fact that the majority of the available methods use event-related information. This is not true, since the majority of cell assembly detection methods indeed do not. I suggest correcting this sentence accordingly.

We thank the reviewer for this comment. Following your advise we have corrected the sentence, highlighting its usefulness, even in the absence of the presentation times of stimuli.

8) All the free parameters used should be available to the reader without forcing him to dig into the code.

As suggested in the previous round of review, the revised version of the manuscript now includes a Table (Table 1) with the parameter's name, a description, and the default value. There are no other parameters in our algorithm.
